# Reflecting on an Intense Year for Transplant International

**DOI:** 10.3389/ti.2022.11118

**Published:** 2022-12-23

**Authors:** Thierry Berney, Maria Irene Bellini, Nuria Montserrat, Maarten Naesens, Thomas Neyens, Stefan Schneeberger

**Affiliations:** ^1^ Editor-in-Chief, Transplant International; ^2^ Deputy Editor-in-Chief, Transplant International; ^3^ Social Media Editor, Transplant International; ^4^ Statistical Editor, Transplant International

The spirit of *Transplant International* is characterized by anticipation of the developments lying ahead and attempt to grow through generated opportunities. In an increasingly digital world and amid growing concerns for the environment, we adopted a paperless format years before our competitors. We have taken advantage of the potential offered by digitalization and designed a journal with a completely novel appearance, with the animated covers as a hallmark (1). Open science, embodied in the FAIR acronym (Findability, Accessibility, Interoperability, Reusability), is not only a self serving principle but has become a requirement from academic institutions to their scholars. Also, and more compellingly, most funding agencies increasingly demand that scientific outputs resulting from public financial support be made freely available (2). We strongly believe that these changes are here to stay and that all scientific research will be published open access in the near future. In line with all these important changes, and to further adapt to these challenging times, we have made our selection of a new publisher and opted to move to gold open access in 2022 (3). This change was very well adopted by the community and resulted in close to 250, carefully peer reviewed, high-quality articles published in regular or special issues in 2022. We would like to thank the authors who have chosen *Transplant International* to publish their cutting-edge research. In this issue, we also wish to highlight the articles that have received the most attention in 2022 and congratulate their authors. Our thanks also go to our executive and associate editors and our reviewers, whose work and expertise are the bedrock of *Transplant International*. Happy New Year and may 2023 bring exciting achievements to all!

**TOP 10 2022 MOST CITED ARTICLES**

**Table:** 

Bio-Engineering of Pre-Vascularized Islet Organoids for the Treatment of Type 1 Diabetes	Wassmer et al.
Immune Response to Third Dose BNT162b2 COVID-19 Vaccine Among Kidney Transplant Recipients—A Prospective Study	Yahav et al.
Machine Perfusion for Human Heart Preservation: A Systematic Review	Qin et al.
BNT162b2 Third Booster Dose Significantly Increases the Humoral Response Assessed by Both RBD IgG and Neutralizing Antibodies in Renal Transplant Recipients	Hod et al.
Sense and Sensibilities of Organ Perfusion as a Kidney and Liver Viability Assessment Platform	Verstraeten et al.
Comparison of mRNA-1273 and BNT162b2 SARS-CoV-2 mRNA Vaccine Immunogenicity in Kidney Transplant Recipients	Haller et al.
Perfusate Composition and Duration of *Ex-Vivo* Normothermic Perfusion in Kidney Transplantation: A Systematic Review	Fard et al.
Surrogate Endpoints for Late Kidney Transplantation Failure	Naesens et al.
Kidney Transplants From Donors on Extracorporeal Membrane Oxygenation Prior to Death Are Associated With Better Long-Term Renal Function Compared to Donors After Circulatory Death	Gregorini et al.
Kidneys for Sale: Empirical Evidence From Iran	Moeindarbari et al.

**TOP 10 2022 HIGHEST ATTENTION ARTICLES (ALTMETRIC SCORES)**

**Table:** 

European Guideline for the Management of Kidney Transplant Patients With HLA Antibodies: By the European Society for Organ Transplantation Working Group	Mamode et al.
Insights From Transplant Professionals on the Use of Social Media: Implications and Responsibilities	Sandal et al.
Kidneys for Sale: Empirical Evidence From Iran	Moeindarbari et al.
Gender and Racial Disparity Among Liver Transplantation Professionals: Report of a Global Survey	Aguilera et al.
Donor Autonomy and Self-Sacrifice in Living Organ Donation: An Ethical Legal and Psychological Aspects of Transplantation (ELPAT) View	Mamode et al.
From Haphazard to a Sustainable Normothermic Regional Perfusion Service: A Blueprint for the Introduction of Novel Perfusion Technologies	Hunt et al.
Criminal, Legal, and Ethical Kidney Donation and Transplantation: A Conceptual Framework to Enable Innovation	Roth et al.
Kidneys for Sale: Are We There Yet?	Jackson et al.
Kidneys for Sale? A Commentary on Moeindarbari’s and Feizi’s Study on the Iranian Model	Ambagtsheer et al.
An Analysis by the European Committee on Organ Transplantation of the Council of Europe Outlining the International Landscape of Donors and Recipients Sex in Solid Organ Transplantation	Cozzi et al.

**TOP 10 2022 MOST DOWNLOADED ARTICLES**

**Table:** 

European Guideline for the Management of Kidney Transplant Patients With HLA Antibodies: By the European Society for Organ Transplantation Working Group	Mamode et al.
Bio-Engineering of Pre-Vascularized Islet Organoids for the Treatment of Type 1 Diabetes	Wassmer et al.
Proposed Definitions of T Cell-Mediated Rejection and Tubulointerstitial Inflammation as Clinical Trial Endpoints in Kidney Transplantation	Seron et al.
Evolution of the Definition of Rejection in Kidney Transplantation and Its Use as an Endpoint in Clinical Trials	Becker et al.
Plasma Biomarkers for Clinical Assessment of Bone Mineral Density in Heart Transplanted Patients—A Single-Center Study at Skåne University Hospital in Lund	Löfdahl et al.
Sense and Sensibilities of Organ Perfusion as a Kidney and Liver Viability Assessment Platform	Verstraeten et al.
Metagenomic Next-Generation Sequencing for Diagnosing Infections in Lung Transplant Recipients: A Retrospective Study	Ju et al.
Prolonged-Release Once-Daily Formulation of Tacrolimus Versus Standard-of-Care Tacrolimus in *de novo* Kidney Transplant Patients Across Europe	Budde et al.
Demonstrating Benefit-Risk Profiles of Novel Therapeutic Strategies in Kidney Transplantation: Opportunities and Challenges of Real-World Evidence	Helanterä et al.
Perfusate Composition and Duration of *Ex-Vivo* Normothermic Perfusion in Kidney Transplantation: A Systematic Review	Fard et al.

**TOP 10 2022 MOST VIEWED ARTICLES**

**Table:** 

Kidneys for Sale: Empirical Evidence from Iran	Moeindarbari et al.
Comparison of mRNA-1273 and BNT162b2 SARS-CoV-2 mRNA Vaccine Immunogenicity in Kidney Transplant Recipients	Haller et al.
European Guideline for the Management of Kidney Transplant Patients With HLA Antibodies: By the European Society for Organ Transplantation Working Group	Mamode et al.
Bio-Engineering of Pre-Vascularized Islet Organoids for the Treatment of Type 1 Diabetes	Wassmer et al.
Kidneys for Sale: Are We There Yet?	Jackson et al.
Cardiac Xenotransplantation: Progress in Preclinical Models and Prospects for Clinical Translation	Singh et al.
From Haphazard to a Sustainable Normothermic Regional Perfusion Service: A Blueprint for the Introduction of Novel Perfusion Technologies	Hunt et al.
Patient Selection for Downstaging of Hepatocellular Carcinoma Prior to Liver Transplantation—Adjusting the Odds?	Seehofer et al.
Mortality and Causes of Death After Liver Transplantation: Analysis of Sex Differences in a Large Nationwide Cohort	Serrano et al.
Donor Autonomy and Self-Sacrifice in Living Organ Donation: An Ethical Legal and Psychological Aspects of Transplantation (ELPAT) View	Mamode et al.

**EDITORS’ PICKS: 2022 COVER ARTICLES**

**Table:** 

January	Bio-Engineering of Pre-Vascularized Islet Organoids for the Treatment of Type 1 Diabetes	Wassmer et al.
February	Prolonged Organ Extraction Time Negatively Impacts Kidney Transplantation Outcome	Maassen et al
March	Cardiac Xenotransplantation: Progress in Preclinical Models and Prospects for Clinical Translation	Singh et al.
April	Patient Selection for Downstaging of Hepatocellular Carcinoma Prior to Liver Transplantation—Adjusting the Odds?	Seehofer et al.
May	A National Survey Comparing Patients’ and Transplant Professionals’ Research Priorities in the Swiss Transplant Cohort Study	Beckmann et al.
June	Kidneys for Sale: Empirical Evidence From Iran	Moeindarbari et al.
July	Artificial Intelligence: Present and Future Potential for Solid Organ Transplantation	Peloso et al.
August	Equity in Transplantation: A Commitment for Progress in Troubled Times	Berney et al.
September	Physical Effects, Safety and Feasibility of Prehabilitation in Patients Awaiting Orthotopic Liver Transplantation, a Systematic Review	Jetten et al.
October	Outcome of COVID-19 in Kidney Transplant Recipients Through the SARS-CoV-2 Variants Eras: Role of Anti-SARS-CoV-2 Monoclonal Antibodies	Papadimitriou-Olivgeris et al.
November	Outcomes After Liver Transplantation With Incidental Cholangiocarcinoma	Safdar et al.
December	Association of DGF and Early Readmissions on Outcomes Following Kidney Transplantation	Jadlowiec et al.

## Appendix

Transplant International thanks the following colleagues for their time and effort in reviewing manuscripts during 2022. Their astute assessments along with their productive and often critical comments ensure that only high-quality papers are published in Transplant International.

**Table:**

David Adra, United States	Nicholas Gilbo, Belgium
Pedro Aguiar, Spain	Gian Grazi, Italy
Clemens Aigner, Germany	Julia Grottenthaler, Germany
Nobuhisa Akamatsu, Japan	Angelika Gruessner, United States
Emin Akin, Turkey	Gaurav Gupta, United States
Ruslan Alikhanov, Russia	Finn Gustafsson, Denmark
Gulbin Altun, Turkey	Fadi Haidar, Switzerland
Luis Andrade, Brazil	Kieran Halloran, Canada
Kenneth Andreoni, United States	Karim Hamaoui, United Kingdom
Miha Arnol, Slovenia	Jerome Harambat, France
Zainab Arslan, United Kingdom	Takashi Harano, United States
Anders Åsberg, Norway	Hermien Hartog, United Kingdom
John Asher, United Kingdom	Dan Harvey, United Kingdom
Brad Astor, United States	Koji Hashimoto, United States
John Aubert, Switzerland	Theresa Hautz, Austria
Jean Augusto, France	Siba Haykal, Canada
Mehmet Avci, Turkey	Bulang He, Australia
Markus Barten, Denmark	Manfred Hecking, Austria
Irene Bello, Spain	Rachel Hellemans, Belgium
David Bennett, Italy	Taizo Hibi, Japan
Frederik Berrevoet, Belgium	Luuk Hilbrands, Netherlands
Berend Beumer, Netherlands	Ibtesam Hilmi, United States
Vaishaly Bharambe, India	Caroline Hoed, Netherlands
Massimo Boffini, Italy	Bart Hoek, Netherlands
Lorena Bogaart, Switzerland	Suk Hong, South Korea
Georg Böhmig, Austria	Martin Howell, Australia
Cecilia Bonazzetti, Italy	Petra Hruba, Czechia
Saskia Bos, United Kingdom	Michael Hsin, China
Katrina Bramstedt, Switzerland	Franz Immer, Switzerland
Julien Branchereau, France	Maria Iranzo, Spain
Gerald Brandacher, United States	Fadi Issa, United Kingdom
Willem Brouwer, Netherlands	Gaurav Jain, United States
Olivier Brugiere, France	Trond Jenssen, Norway
Antonio Bruni, Canada	Jong Jeong, South Korea
Ann Bugeja, Canada	Thomas Jouve, France
Caron Burch, United States	José Junior, Brazil
Patrizia Burra, Italy	Takashi Kanou, Japan
Jasper Callemeyn, Belgium	Moritz Kaths, Germany
Luis Camargo, Brazil	Dixon Kaufman, United States
Riccardo Carlis, Italy	Yvelynne Kelly, Ireland
Linda Cendales, United States	Nicos Kessaris, United Kingdom
Leonardo Centonze, Italy	Hussein Khambalia, United Kingdom
Carlo Ceresa, United Kingdom	Attila Kiss, Austria
Evangelos Cholongitas, Greece	Jenny Kissler, Netherlands
Luigi Cirillo, Italy	Daniela Kniepeiss, Austria
Julian Cisneros, Spain	Mladen Knotek, United Kingdom
Antonio Citro, Italy	Alice Koenig, France
Marco Claasen, Canada	Jan Kříž, Czechia
Elizabeth Cohen, United States	Florence Lacaille, France
Audrey Coilly, France	Stéphanie Lacotte, Switzerland
Lionel Couzi, France	Quirino Lai, Italy
Gonzalo Crespo, Spain	Manon Launay, France
Kristopher Croome, United States	Chih Lee, Taiwan
Francesco D'Amico, Italy	David Leeser, United States
John Dark, United Kingdom	Visnja Lezaic, Serbia
Andre David, Brazil	Tsan Lin, Taiwan
Rajeev Desai, United Kingdom	Sandra Lindstedt, Sweden
Olivier Detry, Belgium	Grant Luxton, Australia
Antal Dezsőfi, Hungary	David Machado, Brazil
Fritz Diekmann, Spain	Umberto Maggiore, Italy
Tomas Dopico, United Kingdom	Manuel Maglione, Austria
Cinthia Drachenberg, United States	Rahul Mainra, Canada
Richard Dumbill, United Kingdom	Lorna Marson, United Kingdom
James Eason, United States	Dominique Martin, Australia
Albino Eccher, Italy	Sara Martinez, Spain
Fabian Echterdiek, Germany	Nuria Masnou, Spain
Thomas Egan, United States	Charlotte Maulat, France
Tarek Elbaz, Egypt	Laura Mazilescu, Germany
Juliet Emamaullee, United States	Marilda Mazzali, Brazil
Eric Epailly, France	Lucas McCormack, Argentina
Michiel Erasmus, Netherlands	Lisa McElroy, United States
Hannah Esser, United Kingdom	Shaheed Merani, United States
François Faitot, France	Manuel Meseguer, Spain
Àlex Favà, Spain	Irena Milaniak, Poland
Carl Fischer-Fröhlich, Germany	Robert Minnee, Netherlands
Alejandro Forner, Spain	Geir Mjøen, Norway
Anna Forsberg, Sweden	Beat Moeckli, Switzerland
Anna Francis, Australia	Michele Molinari, United States
Rossana Franzin, Italy	Martin Montenovo, United States
Maristela Freire, Brazil	Jose Morales, Spain
Jonathan Fridell, United States	Christian Morath, Germany
Frank Friedersdorff, Germany	Maria Morelli, Italy
Tji Gan, Netherlands	Francesc Moreso, Spain
Ilaria Gandolfini, Italy	Lúcio Moura, Brazil
Braulio Garza, Mexico	Istvan Mucsi, Canada
Anand Ghanekar, Canada	Alessandra Mularoni, Italy
Davide Ghinolfi, Italy	Bashoo Naziruddin, United States
Fernando Gil, United Kingdom	Flavia Neri, Italy	
James Neuberger, United Kingdom	Kentaro Noda, United States
Ingvild Nordøy, Norway	Shuji Okahara, Australia
Toshihiro Okamoto, United States	Mihai Oltean, Sweden
Lay Ong, United Kingdom	Yan Ou, United States
Lara Øzbay, Denmark	Kagan Ozer, United States
Duilio Pagano, Italy	Renato Pascale, Italy
Concetta Pasquale, Italy	Concetta Pasquale, Italy
Sandra Pavicevic, Germany	Alvaro Pena, United States
Thamara Perera, United Kingdom	Richard Perez, United States
Davide Piloni, Italy	Jacques Pirenne, Belgium
Robert Pol, Netherlands	Wojciech Polak, Netherlands
Mindaugas Rackauskas, United States	Dirk Raemdonck, Belgium
Axel Rahmel, Germany	Siavash Raigani, United States
Raja Ramachandran, India	Pablo Ramírez, Spain
György Reusz, Hungary	Michael Rickels, United States
Peter Riddell, Ireland	Juliano Riella, United States
Renato Romagnoli, Italy	Gianluca Rompianesi, Italy
Christopher Salerno, United States	Luca Santo, Italy
František Saudek, Czechia	Stefan Schaub, Switzerland
Ivo Schurink, Netherlands	Sameep Sehgal, United States
Gennaro Selvaggi, United States	Markus Selzner, Canada
Mehmet Sever, Turkey	Adnan Sharif, United Kingdom
Amit Sharma, United States	Ahmed Sherif, United Kingdom
Lena Sibulesky, United States	Tara Sigdel, United States
Mateusz Soliński, Poland	Goce Spasovski, North Macedonia
Nicola Stefano, Italy	Seiichiro Sugimoto, Japan
Kazunari Tanabe, Japan	Ekamol Tantisattamo, United States
Marta Tejedor, Spain	Gianpaolo Tessari, Italy
Hoyee Tiong, Singapore	Burkhard Tönshoff, Germany
Meng Tsai, Taiwan	Cynthia Tsien, Canada
Maarten Tushuizen, Netherlands	Tuerhongjiang Tuxun, China
Acar Tüzüner, Turkey	Andreas Tzakis, United States
Ivo Tzvetanov, United States	Nicole Valenzuela, United States
Massimiliano Veroux, Italy	Deborah Verran, Australia
Laila Viana, Brazil	Graziano Vignolini, Italy
André Vincentelli, France	Arturas Vinikovas, Lithuania
Robin Vos, Belgium	Johan Walle, Belgium
Charles Wassmer, United States	Annelies Weerd, Netherlands
Emmanuel Weiss, France	Colin Wilson, United Kingdom
Tineke Wind, Netherlands	Anna Winterbottom, United Kingdom
Dafna Yahav, Israel	Masahiko Yazawa, Japan
Baris Yildiz, Turkey	Kenneth Yong, Australia
Alan Zhuang, United Kingdom	Emanuel Zitt, Austria
Martina Zwaan, Germany	Bartlomiej Zych, United Kingdom
